# Giant Magnetostriction in Ferrimagnetic SmFe_5_As_3_

**DOI:** 10.1002/anie.202522578

**Published:** 2026-03-25

**Authors:** Oksana Karychort, Jan Priessnitz, Volodymyr Buturlim, Mitja Krnel, Iñigo Robredo, Nazar Zaremba, Ulrich Burkhardt, Helge Rosner, Andrew Fitch, Markus König, Fabrice Wilhelm, Olha Zhak, Dileep Krishnan, Ivan Soldatov, Rudolf Schäfer, Maia Vergniory, Libor Šmejkal, Theo Siegrist, Berit H. Goodge, Krzysztof Gofryk, Yurii Prots, Eteri Svanidze

**Affiliations:** ^1^ Max Planck Institute for Chemical Physics of Solids Dresden Germany; ^2^ Department of Analytical Chemistry Ivan Franko National University of Lviv Lviv Ukraine; ^3^ Max Planck Institute for the Physics of Complex Systems Dresden Germany; ^4^ Idaho National Laboratory Idaho Falls Idaho USA; ^5^ Luxembourg Institute of Science and Technology (LIST) Esch‐sur‐Alzette Luxembourg; ^6^ European Synchrotron Radiation Facility (ESRF) Grenoble France; ^7^ Thermo Fisher Scientific Eindhoven The Netherlands; ^8^ Leibniz Institute for Solid State and Materials Research Dresden (IFW Dresden) Dresden Germany; ^9^ Regroupement Québécois sur les Matériaux de Pointe (RQMP) Québec Canada; ^10^ Département de Physique et Institut Quantique Université de Sherbrooke Sherbrooke Québec Canada; ^11^ Institute of Physics Czech Academy of Sciences Prague Czech Republic; ^12^ Department of Chemical and Biomedical Engineering FAMU–FSU College of Engineering Florida State University Tallahassee Florida USA; ^13^ National High Magnetic Field Laboratory Tallahassee Florida USA

**Keywords:** crystal structure, functional materials, magneto‐elastic coupling, magnetostriction, negative thermal expansion, novel ferrimagnetic arsenides, zero thermal expansion

## Abstract

Magnetostrictive materials are of interest not only from a fundamental perspective but also for their potential applications, spanning spintronics to energy harvesting. A new magnetostrictive material—SmFe_5_As_3_—reveals a complex interplay between magnetostriction, magnetic properties, and the crystal structure behavior. The ground state of SmFe_5_As_3_ is ferrimagnetic, as evidenced by magnetic susceptibility data, band‐structure calculations, and XANES measurements. At $T_{m1}=28\pm 4$ K, part of the Fe sublattice reorients, transitioning into a ferromagnetic state. This is followed by an entrance into the paramagnetic state at $T_{m2}=76\pm 4$ K. The effects observed in the magnetic measurements are accompanied by structural phase transitions. All three phases—below $T_{m1}$, between $T_{m1}$ and $T_{m2}$, and above $T_{m2}$—are described by the same structural motif of the UCr_5_P_3_ type (monoclinic space group $P2_{1}/m$), differing only in the degree of deformation of the Fe–As framework. The new material exhibits giant magnetostriction of $2500\times 10^{-6}$. Dilatometry measurements on a SmFe_5_As_3_ single crystal indicate strongly diverse behavior, exhibiting not only negative, but also zero, as well as positive thermo‐elastic effects.

## Graphical abstract

SmFe_5_As_3_ is a newly discovered ferrimagnetic material that displays peculiar volumetric effects as a function of temperature. As a result of two magnetic sublattices—one with d‐electrons of Fe and another with f‐electrons of Sm—single crystals of SmFe_5_As_3_ show negative (blue), positive (pink), and zero (beige) thermal expansion along the [010] axis of the needle‐shaped crystal. The most notable region of giant thermal expansion occurs for 42≤T≤100K, reaching values of up to $2500\times 10^{-6}$.

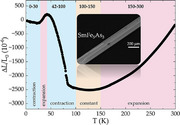

## 1 Introduction

Magnetostriction is the reason why transformers hum—the AC current passing through the primary coil creates a changing magnetic flux in the iron–silicon core, leading to a periodic expansion and contraction of the core sheets in the rhythm of the applied field, thus creating mechanical vibrations. This “singing” depends on the AC frequency, which is country‐specific, and even though the movement is on the order of micrometers, the frequency is doubled because the core stretches on both positive and negative cycles. Iron (or iron–silicon) by itself is moderately magnetostrictive, reaching relative strains of the order of 20–$30\times 10^{-6}$. This is just one example of magnetostrictive materials and the role they play in everyday life—but despite how common they are, their microscopic chemical features are not yet fully understood. When the search for magnetostrictive materials began in the 1970s, the idea of combining iron with rare‐earth elements was quickly recognized, since elements like Dy and Tb are much more magnetostrictive [1, 2]. This led to the discovery of Terfenol‐D (Tb_0.3_Dy_0.7_Fe_2_, 2000–$2500\times 10^{-6}$) – the classic example of a room‐temperature magnetostrictive material [1, 2, 3, 4, 5, 6], which is widely used in technological applications such as actuators, sensors, and transducers [7, 8, 9, 10]. Tb_0.3_Dy_0.7_Fe_2_ crystallizes in the cubic Laves phase C15, exhibiting anisotropic magnetostriction, with the strain in the [111] direction being the largest. Isostructural PrFe_1.9_ and HoFe_2_ also exhibit giant magnetostriction, albeit at cryogenic temperatures ($6700\times 10^{-6}$ for 30 K ≤T≤70 K in Pr and $745\times 10^{-6}$ at T=4.2 K in Ho) [11, 12, 13]. The SmFe_2_ system can reach a strain of $1000\times 10^{-6}$ in H=1 T at room temperature [14]. In general, in many Laves phases containing d‐ and f‐electrons, both magnetostrictive and magnetocaloric effects have been observed as a result of strong magneto‐elastic coupling [15]. Overall, while Terfenol‐D and its derivatives are well integrated into the landscape of materials with large magnetostriction, it remains desirable to find new materials—both from a fundamental and an applied perspective. For example, cheaper alternatives, even for cryogenic applications, are needed [6, 16].

In this work, we present a new material that combines 3d and 4f magnetic species to produce giant magneto‐elastic response. SmFe_5_As_3_ is air‐stable and can be grown in millimeter‐sized single crystals, forming in the UCr_5_P_3_ structure type [17]. In this new material, the flexibility of the Fe–As network, coupled with magnetic Fe and Sm sublattices, leads to the emergence of diverse thermo‐elastic response. SmFe_5_As_3_ is the first representative of this structure type containing two ferrimagnetically coupled sublattices—other members are UCr_5_P_3_[17], UFe_5_As_3_ [18], ZrCr_5_P_3_ [19], and SrIn_4.65_Au_3.35_ [20]. This, perhaps, suggests that exploring modifications of this flexible structural network with rare‐earth elements carrying larger magnetic moments could reveal even more peculiar behavior. Overall, complex interplay between deformation, magnetism, and crystal structure of SmFe_5_As_3_ give insight into how new materials with functional thermo‐elastic properties can be designed and improved.

## 2 Results and Discussion

### 2.1 Synthesis

**FIGURE 1:**
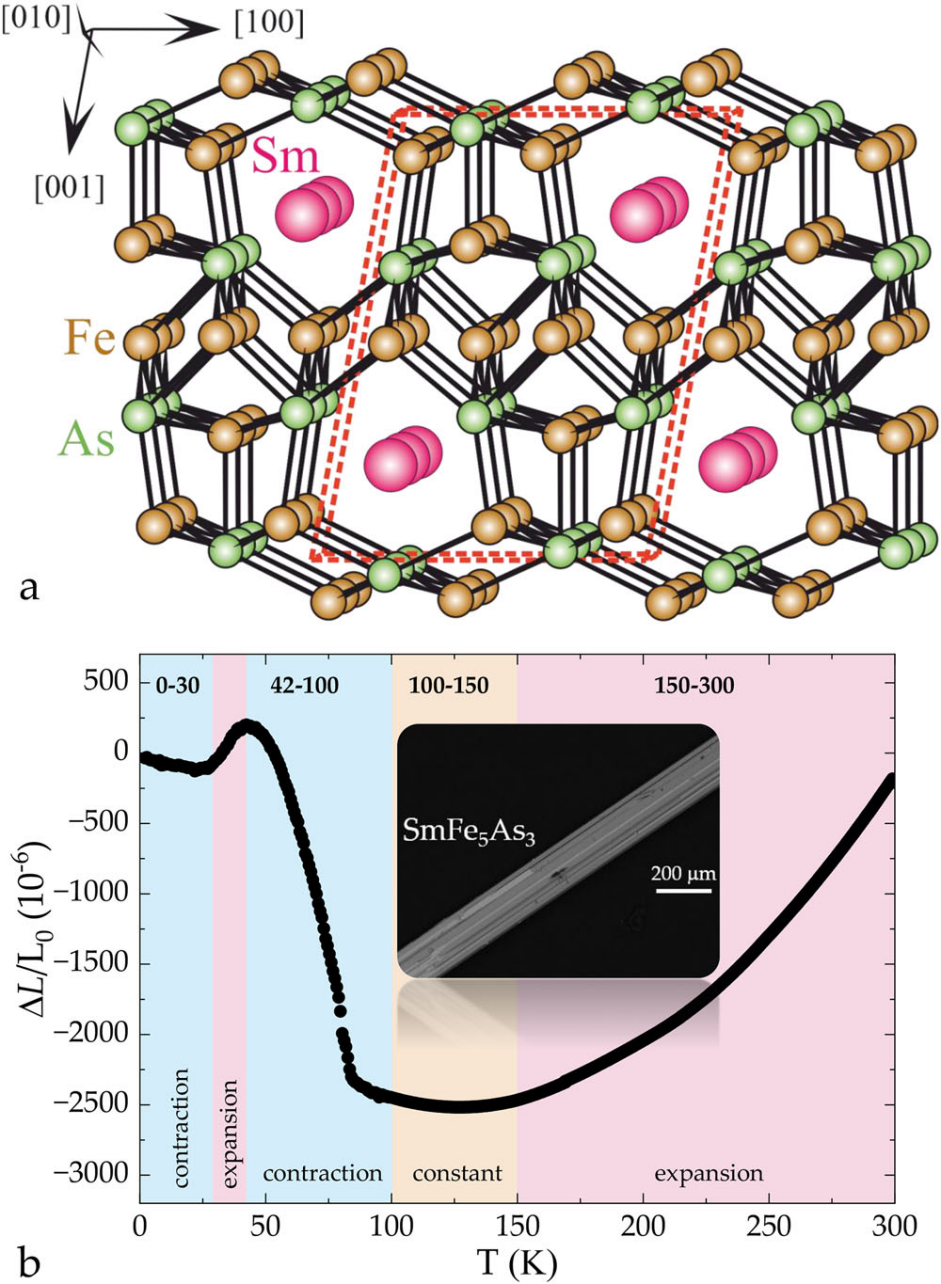
Crystal structure and coexistence of two magnetic species give SmFe_5_As_3_ its unique properties: (a) The crystal structure can be represented as a 3D network of interconnected Fe (brown) and As (green) atoms (d 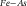 = 2.37–2.62 Å). (b) Peculiar volumetric effects along [010] as a function of temperature—SmFe_5_As_3_ is showing negative, zero, as well as positive thermal expansion for various temperature ranges. Inset: A back‐scattered electron image of a SmFe_5_As_3_ single crystal with the long edge corresponding to the [010] direction.

In the Sm–Fe–As ternary system, only SmFe_4_As_12_ has been reported previously, entering a ferrimagnetic state below T=29 K [21]. It is important to note that the SmFe_4_As_12_ synthesis is not straightforward and requires application of simultaneous high‐temperature (1171 K) and high‐pressure (∼4 GPa), which makes our discovery of a new compound in this ternary space fairly surprising. The new arsenide SmFe_5_As_3_ crystallizes in the monoclinic $P2_{1}/m$ space group, as depicted in Figure 1a. Millimeter‐sized single crystals, shown in Figure 1b, were synthesized by employing bismuth as a flux—similar to the recipe previously used for UFe_5_As_3_ [18]—see Supplementary Information for further details. Some residual bismuth was found to remain on the surface of SmFe_5_As_3_ crystals, but aside from the resistivity measurements, its effect was minimal. Needle‐like single crystals that grow along the [010] direction have a strong tendency to form multi‐twin agglomerates, which made it challenging to examine the crystal structure—see Supplementary Information for more details.

**FIGURE 2:**
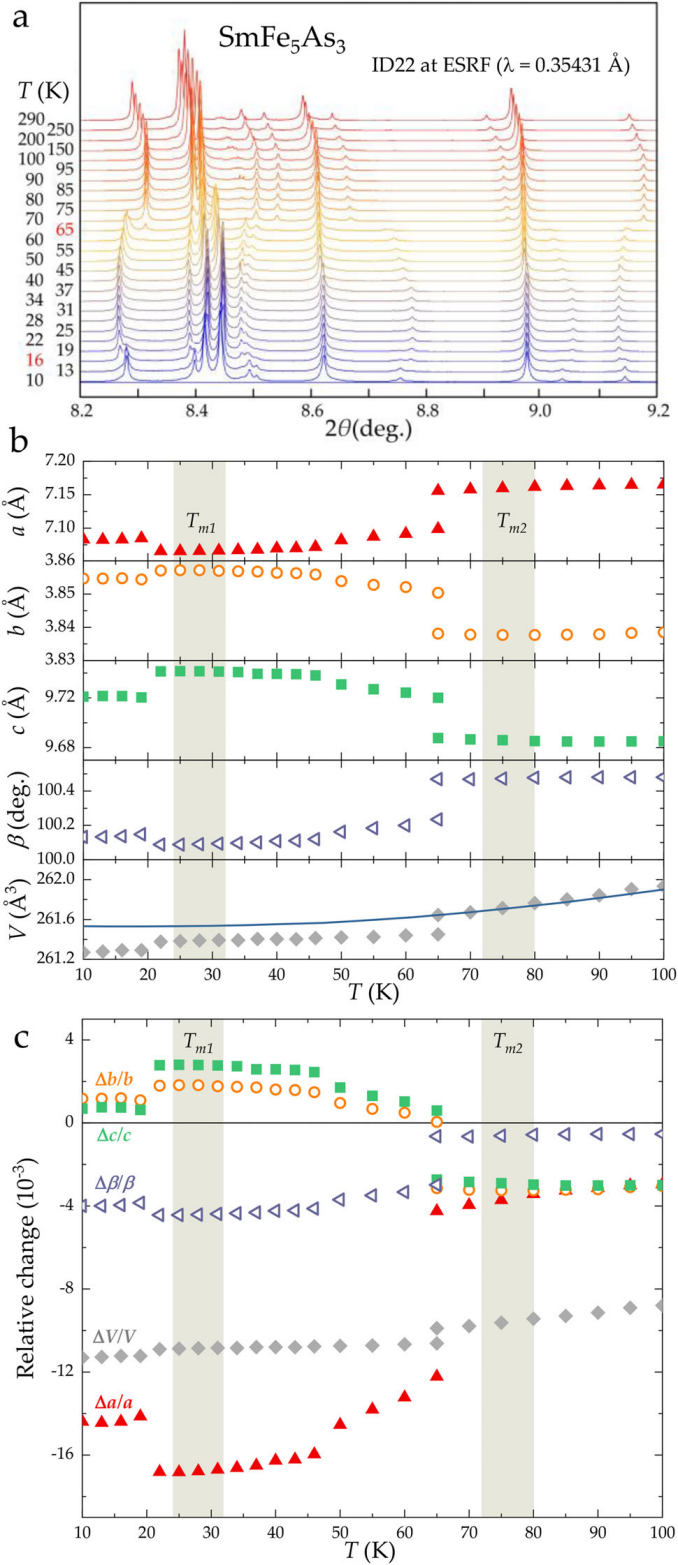
Structural evolution of the SmFe_5_As_3_ with temperature: (a) Selected region of the diffraction patterns, recorded between T=10 K and T=290 K. The temperatures at which two phases coexist are highlighted in red. (b) The behavior of the lattice parameters as a function of temperature with shaded regions marking the magnetic transitions $T_{m1}$ and $T_{m2}$. The blue solid line is a hypothetical lattice behavior obtained by the Grüneisen approximation for non‐magnetic SmFe_5_As_3_ (see Supplementary Information). (c) Relative changes in the lattice parameters illustrate discontinuous first order transition and large anisotropy in the structure of SmFe_5_As_3_. A clear offset in the position of the anomalies in the lattice parameters, compared to the temperatures of the magnetic transition $T_{m1}$ and $T_{m2}$, is a result of sample heating by the synchrotron radiation.

**FIGURE 3:**
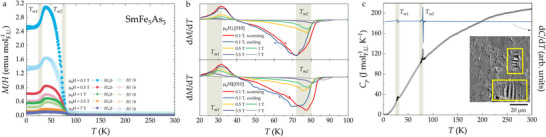
Magnetic behavior of SmFe_5_As_3_: (a) Two transitions are observed in temperature‐dependent susceptibility data for both H∥b (lighter colors, empty symbols) and H⊥b (darker colors, full symbols). Pronounced anisotropic magnetic behavior suggests that ac plane is the easy one. (b) A slight hysteresis is observed in dM/dT for measurements with H∥[010] (red and blue curves in the bottom panel). (c) Both magnetic transition of SmFe_5_As_3_ are evident from the specific heat data. Inset: Magnetic domains observed on the surface of a single crystal cluster at T=50 K.

The morphology of the SmFe_5_As_3_ crystals dictates how the thermo‐electic behavior of this system can be probed. It is clear from Figure 1b, the dilatometric behavior of SmFe_5_As_3_ is unconventional. This is driven, on one hand, by non‐monotonous evolution of the lattice parameters (Figure 2) and, on the other hand, presence of two magnetic transitions (Figure 3). Below, we present a detailed analysis of both sides of this coin—in order to understand magneto‐elastic coupling in this complex material. Overall, the thermo‐elastic behavior of SmFe_5_As_3_ is clearly nontrivial, displaying not only positive, but also negative, as well as zero thermal expansion for different temperature ranges—see Figures 1b and 5.

**FIGURE 4:**
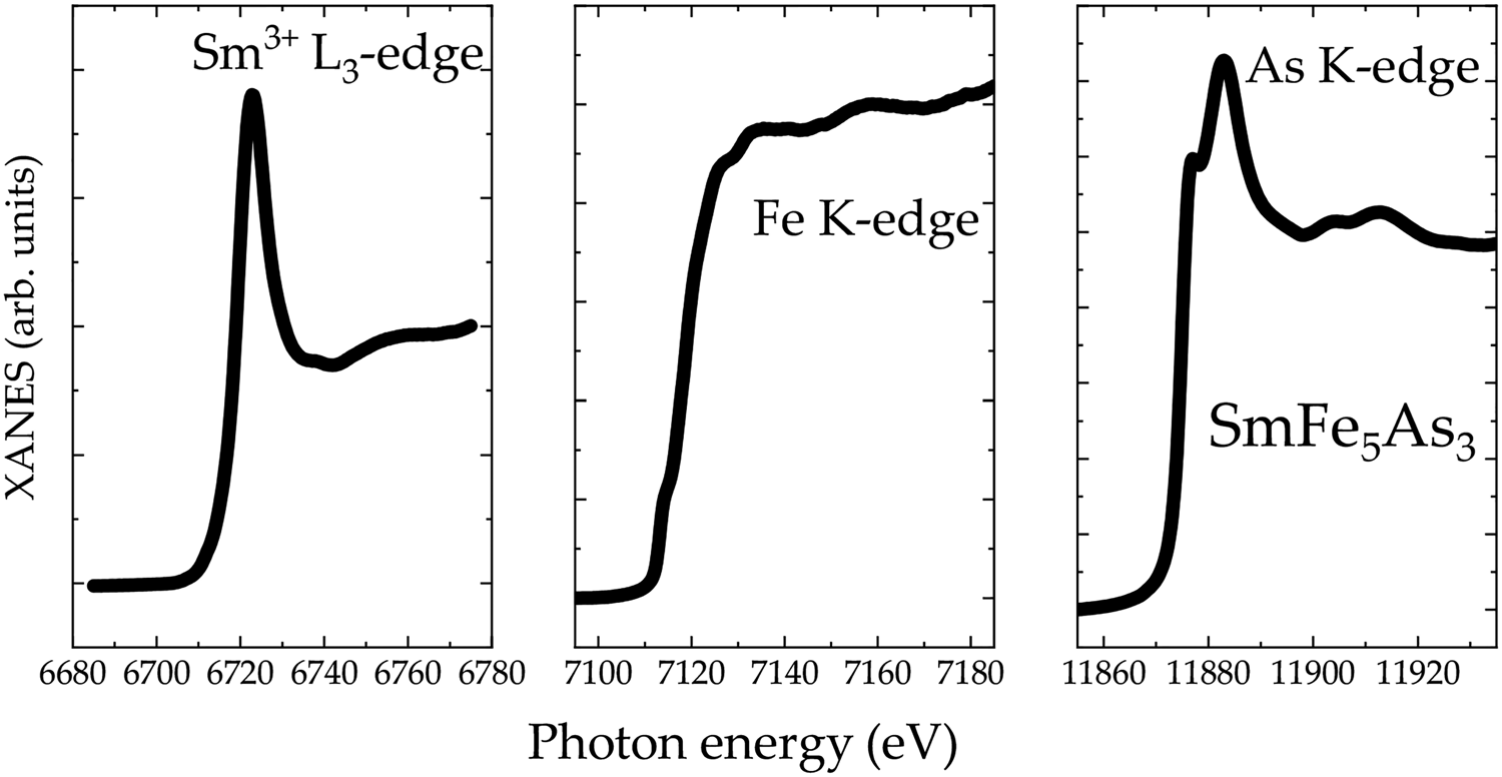
X‐ray absorption near‐edge structure (XANES) analysis: The normalized Sm L_3_‐, Fe K‐ and As K‐egde XANES spectra of SmFe_5_As_3_, measured at T=300 K. The ${\mathrm{Sm}}^{+3}$ state is revealed, while the Fe spectra suggest its itinerant magnetic character.

**FIGURE 5:**
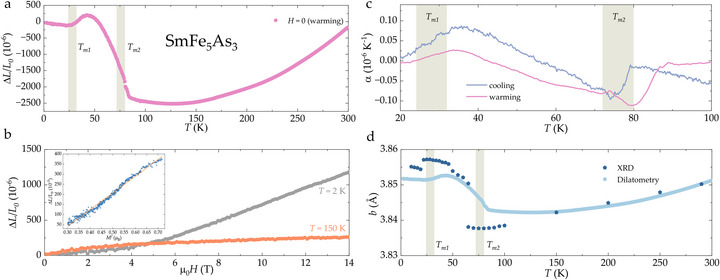
Thermo‐ and field‐elastic properties of SmFe_5_As_3_: (a) Relative change in the length of the the crystal (measured along the b‐axis) reaches $2500\times 10^{-6}$ at T=80 K. (b) Linear thermal expansion $\Delta L/L_{0}$ of single‐crystalline SmFe_5_As_3_, measured at 2 and 150 K parallel to [010] direction as a function of longitudinal magnetic field. Inset: the magnetostriction versus the square of magnetization (see text). (c) A significant difference exists between the position of the maximum on cooling (blue) and on warming (pink), especially for the upper anomaly. (d) Temperature dependence of the lattice parameter *b*, obtained from the x‐ray diffraction (dark blue) and dilatometry (light blue) measurements.

### 2.2 Crystal Structure

The new compound SmFe_5_As_3_ belongs to a family of layered intermetallic systems characterized by a metal‐to‐metalloid ratio of 2:1. The crystal structures of this series are often described as an arrangement of trigonal prisms around the main group atoms. In the structure of SmFe_5_As_3_, trigonal prisms composed of [Sm_2_Fe_4_] units are formed around single As atom. Distinctive shamrock‐like structural motifs emerge from the shared Sm–Sm edge of these metal‐based prisms. These motifs are connected by shared Fe–Fe edges, forming continuous chains along the a axis. The neighboring chains are shifted relative to each other by 1/2 of the shortest lattice translation period b. A more detailed analysis of the shamrock‐like atomic arrangement characteristic of rare‐earth and transition‐metal arsenides with a 1–5–3 composition has been reported in the discussion of the isotypic crystal structure of UFe_5_As_3_[18], as well as U_8_Co_42_As_25_ [22].

Crystal structure of SmFe_5_As_3_ was determined from single crystal data by using a twinned specimen formed by two domains—atomic resolution high‐angle annular dark‐field (HAADF) scanning transmission electron microscopy (STEM) investigation clearly shows these domains with well‐defined growth boundaries (Figure S5). The formation of twins is probably caused by the pseudo‐trigonal motif of the iron sublattice (Figures 1, S5, and S4). With this information, it was relatively easy to identify the distribution of individual domains in the rather complex image of the reciprocal space, reconstructed from a single crystal experiment (more details are given in the Supplementary Information). Interestingly, in SmFe_5_As_3_ (Figure S5), twinning appears to have only a mild effect on the magnitude of the magnetostriction anomaly—see Figure 5d. The lattice parameter b values, extracted from dilatometry (light blue) and x‐ray diffraction (dark blue) data, follow the same trend.

An analysis of the interatomic distances (Table S3 reveals that closest Fe–As contacts (2.375–2.479 Å) are slightly above the sum of the covalent radii of these elements ($r_{Fe+As}$ = 2.37 Å). Taking this into account, the crystal structure of SmFe_5_As_3_ can be described as a 3D Fe–As framework, formed around Sm atoms. The closest homonuclear contacts between Fe atoms occur in the range of 2.601–2.737 Å. These values are larger than the distances observed in elemental Fe (2.48 Å). Viewed from another perspective, the Fe–Fe contacts in SmFe_5_As_3_ are comparable to those recently reported for Th_2_Fe_12_As_7_ [23]. The bonding analysis carried out for Th_2_Fe_12_As_7_ revealed that large separation of Fe atoms precludes magnetic order—this system remains paramagnetic [23]. This suggests that magnetism in SmFe_5_As_3_ is not only driven by Fe, but rather by both magnetic species present in the structure. It is noteworthy that the number of homonuclear neighbors around the Fe atoms vary between 2 and 5. The latter are observed for the Fe2 and Fe4 positions, which are located in a zig‐zag arrangement at *z* = 1/2 along the [100] direction. It can be assumed that this part of the structure contributes significantly to the structural deformation of the Fe–As framework, driven by the rearrangement of magnetic moments around magnetic transitions. The Sm–Fe and Sm–As distances show significantly higher values, compared to the sum of covalent radii (r 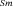 + $r_{Fe}$ = 2.87 Å  and $r_{Sm}$ + $r_{As}$ = 2.82 Å). The shortest Sm–Sm distance (3.8505(9) Å) corresponds to the shortest period b and is slightly larger those observed in elemental Sm (3.579 Å).

The powder X‐ray diffraction data, recorded between 10 and 290 K reveal two first order transitions around 20 and 65 K, which correspond to the temperatures $T_{m1}$ and $T_{m2}$, also present in the measurements of magnetic properties, described below. The temperature of the transformation of the crystal structure differs slightly from the anomalies corresponding to the magnetic transitions—see the shaded regions in Figure 2b,c. This difference is probably due to the heating of the sample by the intense synchrotron source, which is more pronounced at lower temperatures. This is why the phase transition at T∼30 K was initially practically undetectable—when the conventional set‐up of the beamline is used. Only after reducing the intensity of the beam by means of an attenuator, we were able to detect a discontinuous development of the reflection positions at low temperatures, around the phase transition $T_{m1}$ (see further details in the Supporting Information).

**FIGURE 6:**
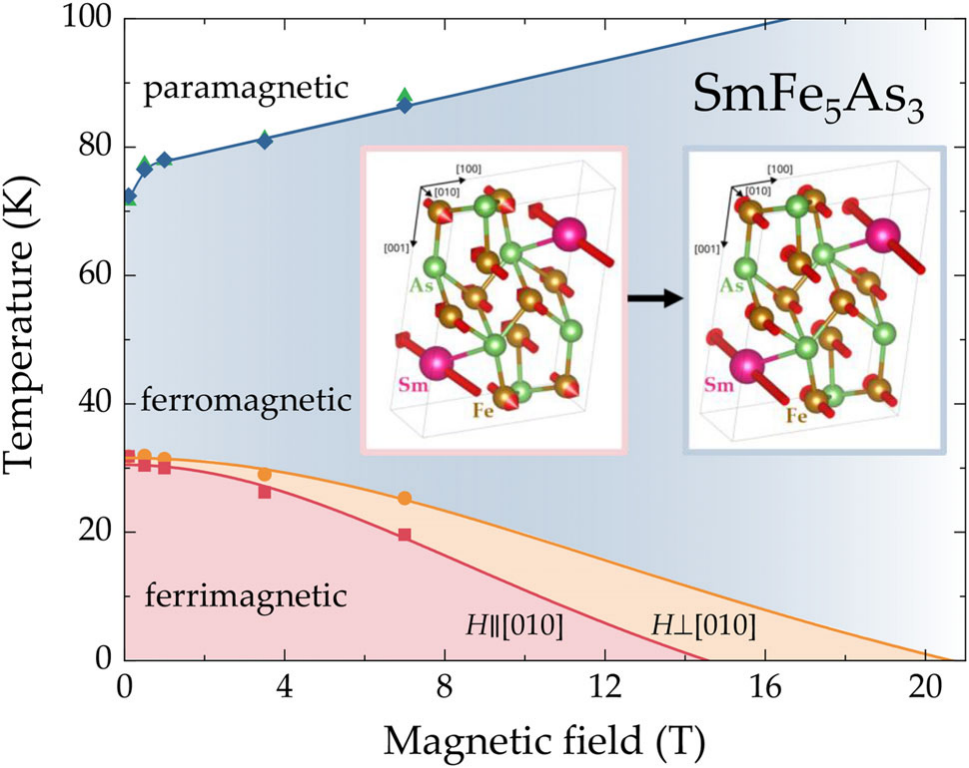
The magnetic phase diagram of SmFe_5_As_3_ reveals three distinct regions—ferrimagnetic (pink and orange), ferromagnetic (blue), and paramagnetic (white). Lines are guides to the eye. The field with which $T_{m1}$ can be suppressed depends on the orientation—for H∥b, the value is lower, consistent with b being the hard magnetic axis. The value of the critical field for H⊥b is, consequently, larger (orange). The two magnetic configurations are shown in the inset.

It is important to note that all three phases detected for SmFe_5_As_3_ (below 20 K, between 20 and 65 K, above 65 K) are identical from crystallographic point of view. However, the presence of discontinuity in the evolution of lattice parameters and the coexistence of two phases in the transition temperature regions allows us to classify these phases as individual modifications, even though they have identical crystallographic attributes such as space group and structural model. It is likely that the observed phase transitions are caused by the anisotropic deformation of the Fe–As network, as a result of spin reorientation from ferri‐ to ferro‐ and to paramagnetic configurations—see Figure 6a,b. To compare, we have also included a hypothetical lattice change of SmFe_5_As_3_ in the absence of the magnetic ordering, as shown by the solid blue line. This was obtained by fitting the second‐order Grüneisen approximation [24, 25, 26, 27, 28, 29] to the ΔV/V in the paramagnetic state using the parameters: $V_{0}$ = 261.53 Å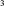 and $\theta_{D}$ = 311 K (see also Supplementary Information for more details). The difference between the extrapolated Grüneisen dependence in the magnetically ordered region and the measured thermal expansion can be associated to the spontaneous exchange magnetostriction.

Given inability of accurately resolving the exact temperatures for $T_{m1}$ and $T_{m2}$ from the x‐ray powder diffraction experiments, we instead employ thermodynamic measurements. Both magnetic transitions of SmFe_5_As_3_ are marked by sharp λ‐type anomalies in the specific heat data, occurring at T=29 K and T=80 K (Figure 3c). The derivative of the resistivity data indicate transitions around T=24 K and T=77 K (Figure S7e), respectively, while derivative of temperature‐dependent susceptibility yield T=32 K and T=72 K (Figure 3b). These three measurements—typically used to establish the ordering temperature of a given material—provide an estimate of $T_{m1}=28\pm 4$ K and $T_{m2}=76\pm 4$ K for SmFe_5_As_3_, which are marked by shaded regions in the relevant figures. For the rest of the discussion, we therefore refer to $T_{m1}$ and $T_{m2}$ as the temperatures of the magnetic transitions, determined from magnetization, specific heat, and resistivity data.

### 2.3 Magnetic Properties

Magnetic susceptibility of SmFe_5_As_3_, shown for the two crystallographic directions in Figure 3a, clearly indicates that SmFe_5_As_3_ is anisotropic, with the ac being the easy plane. This is also clear from the shape of the magnetic domains, shown in the inset of Figure 3c. According to the values of the effective magnetic moments—together with the XANES data showing that Sm is in the +3 oxidation state—see the Figure 4—we postulate that both Sm and Fe species carry local moment in SmFe_5_As_3_. Based on the shape of the magnetic susceptibility, magnetic isotherms (see Figure S7), and results of the band structure calculation (see below), we predict that the ground state of SmFe_5_As_3_ is ferrimagnetic, with all moments being collinear in the [010] direction. More precisely, the most energetically favorable configuration—according to the surveyed results—indicates that two of the Sm and six of the Fe moments are co‐aligned, while four Fe moments are anti‐aligned, see Figure 6a. Of course, the magnetic structure of SmFe_5_As_3_ is fairly complex, as a result of two magnetic species present, which is why a more detailed analysis of the exact magnetic configuration of this material remains a pertinent topic for a future study.

As presented in Figure 3b, strong anomaly accompanies entrance into magnetically ordered state at $T_{m2}=76\pm 4$ K, with a less pronounced feature around $T_{m1}=28\pm 4$ K. This is consistent with the evolution of the lattice parameters, shown in Figure 2c, which indicates that a much bigger lattice change is observed for the upper magnetic transition, namely at $T_{m2}$. Both magnetic transitions are affected by application of an external magnetic field—the value of $T_{m1}$ is decreased, while that of $T_{m2}$ is increased with increasing magnetic field. This allows to construct a magnetic phase diagram for SmFe_5_As_3_, shown in Figure 6a. To further assess the oxidation state of the Sm ions, the x‐ray absorption near‐edge structure (XANES) around the Sm L_3_ absorption edge was recorded—see Figure 4. At room temperature, the intense “white line” corresponding to dipole‐allowed 2p→5d,6s transitions has a maximum at a photon energy of 6723 eV. This is commensurate with an oxidation state assignment of ${\mathrm{Sm}}^{+3}$ [30], which is a quantum system 4$f^{5}$ with L=3 and *S* = 5/2 being magnetic in SmFe_5_As_3_. The spectral shape of the XANES at Fe K‐edge shows a metallic character since there is no pre‐peak, indicative of ionic character. Therefore, the magnetism of the Fe is mostly delocalized—consistent with the magnetization data. Regarding the As K‐edge, it has a typical absorption edge with an the absorption jump feature (11877 eV) that has a predominant As 4p character with admixed Sm d whereas another feature (11883 eV) is due to As 4p admixed with Fe p states.

From the Curie–Weiss fitting, performed for the temperatures above the magnetic transitions (i.e. T>125 K), a negative Weiss temperature on the order of $T_{m1}$ is obtained (Figure S7a,b). The absolute magnitude is larger for the H⊥[010], indicating that the antiparallel sublattice interaction is stronger within the ac plane. The estimated effective magnetic moments range from 7.95 to 10.4 $\mu_{B}$ per formula unit (FU). Given that the value of the effective moment for Fe is between 4.9 and 5.9 $\mu_{B}$ and that for Sm is 0.85 $\mu_{B}$, it is likely that some of Fe is itinerant. Based on the respective sizes of the saturated and effective magnetic moments, extracted from magnetic isotherms, the value of the Rhodes–Wohlfarth ratio [31] for SmFe_5_As_3_ can be estimated. For both orientations, the ratio is above 1, namely 8.8 for H∥b and 4.4 for H⊥b. This suggests that the $T_{m2}=76\pm 4$ K transition is likely itinerant in nature (and is driven by the moments of Fe), similar to what has been reported for structurally related La_12_Fe_57.5_As_41_ [32]. The specific heat data, shown in Figure 3, exhibits characteristic sharp anomalies around $T_{m1}$ and $T_{m2}$. In principle, the analysis of entropy for these transitions would have helped to clearly differentiate between itinerant and local scenarios of SmFe_5_As_3_. However, since we were not able to synthesize an isostructural non‐magnetic analogue, coupled with high ordering temperature of SmFe_5_As_3_, a meaningful phonon analysis could not be performed. This calls for further investigations of the magnetism in SmFe_5_As_3_ using μSR or neutron diffraction, which is certain to provide valuable insights into its precise magnetic character.

### 2.4 Thermo‐ and Field‐Elastic Behavior

The discussion above highlights that magnetism in SmFe_5_As_3_ arises from a complex interplay between Fe and Sm magnetic species. This leads to a nontrivial temperature dependence of the linear thermal expansion, $\Delta L/L_{0}\left(T\right)$, and an exceptionally large magnetostriction ($\Delta L/L_{0}\left(T\right)$ ∼ 2500 ppm) for 42≤T≤100K—see Figure 5. As seen from Figure 5a, the $\Delta L/L_{0}$ curve decreases with decreasing temperature from 300 K, as expected for most materials [33], until it reaches the magnetic phase transition at $T_{m2}$. Below this temperature, a negative thermal expansion (NTE) is observed for the [010] direction [34]. NTE is a rare thermodynamic phenomenon in which materials expand upon cooling, contrary to typical behavior. Such materials have garnered increasing attention due to their potential applications in precision devices such as mechanical components, circuit boards, optical fiber gratings, and high‐precision mirrors [35, 36].

Another anomaly in $\Delta L/L_{0}\left(T\right)$ appears at $T_{m1}$—see Figure 5. These features are more clearly resolved in the thermal expansion coefficient, α(T), shown in Figure 5c, which also reveals hysteresis. A similar hysteresis around $T_{m1}$ was also observed in the x‐ray powder diffraction patterns, as well as magnetic susceptibility (Figure 3b). Notably, α(T) closely follows the temperature derivative of magnetization dM/dT, indicating that the lattice of SmFe_5_As_3_ is strongly coupled to the magnetic entropy via magneto‐elastic interactions. Since the analysis of the phonon contribution to the specific heat could not be carried out (see discussion above), we are also not able to fully quantify the behavior of the SmFe_5_As_3_ lattice in the absence of magnetic transitions. We are, however, certain that the behavior of SmFe_5_As_3_ highlights that thermal expansion in this material is magnetically driven, with the lattice effectively “reading out” the magnetic entropy, as a result of strong magneto‐elastic coupling. Field‐dependent measurements of $\Delta L/L_{0}$ along the b‐axis at T=2 K and 150 K (Figure 5b) further support this conclusion. At T=2 K, the magnetostriction reaches values as high as 1200 ppm at H=14 T, consistent with a significant magneto‐elasticity/magneto‐volumetric effect. As mentioned above, dilatometric response was only probed along the long axis of the crystal, that is, b‐axis, for which the extracted values agree with those estimated from the diffraction data—see Figure 5d. To highlight the intrinsic magnetoelastic response, we replot the forced magnetostriction as a function of the magnetization, ΔL/L(M), rather than the applied field (see the inset of Figure 5b). In the magnetic field range above 2 T, the data show an approximately linear dependence when plotted versus $M^{2}$, consistent with the lowest‐order symmetry‐allowed magnetoelastic coupling, $\Delta L/L=a+bM^{2}$, where a is a field‐independent offset and b parameterizes the effective magnetoelastic coupling for the measured strain direction. This quadratic dependence reflects the leading magnetoelastic term allowed by time‐reversal symmetry, which requires the strain to be even in M [37, 38, 39]. The near‐linearity of ΔL/L versus $M^{2}$ indicates that the dominant contribution to the forced magnetostriction arises from this even‐in‐M term. Any systematic deviations from the relation above would suggest higher‐order contributions, for example, $\Delta L/L=a+bM^{2}+cM^{4}$, or a field‐driven evolution of the magnetic state.

It is important to note that the relative change in the lattice parameter a is the largest – see the red symbols in Figure 2c. It is therefore reasonable to expect that the magnetostrictive effect reported in this work along the needle's b‐axis is not the largest that, in principle, is possible in the SmFe_5_As_3_ compound. The experimental determination of the dilatometric behavior along the [100] axis was made impossible by the extremely small dimensions of SmFe_5_As_3_ crystals in this direction, that is, thickness of the needle on the order of 200 μm. This, in principle, suggests that if the SmFe_5_As_3_ compound can be grown in a larger single crystal form or with a different sample morphology (either in bulk or in thin film form), even more impressive thermo‐elastic behavior can potentially be achieved, especially for the temperatures below $T_{m1}$.

### 2.5 Band Structure Calculations

In order to further understand the microscopic magnetism of SmFe_5_As_3_, ab initio density functional theory (DFT) calculations were carried out. First, DFT was employed to explore the magnetic configuration space and predict the ground state in terms of local magnetic moments within the unit cell. In the unit cell containing 12 magnetic sites, all $2^{12}=4096$ collinear magnetic configurations (representing all possible combinations of up/down moment directions) were used as initial states for individual calculations. All calculations converged to several stable magnetic configurations. The configuration with the lowest energy corresponds to a ferrimagnetic state. The converged ground state is collinear up to minuscule deviations of the magnetic moments, caused by limited DFT precision. The moments are aligned along the b‐axis, though the extremely small energy scale of the magnetic anisotropy forbid us to establish a more definitive figure.

The crystal structure of SmFe_5_As_3_ is described in the monoclinic symmetry with space group $P2_{1}/m$ (no. 11). All 18 atoms in the unit cell are arranged as pairs in 9 independent 2e Wyckoff positions—see inset of Figure 6. Five independent Fe positions indicate that a more complicated (ferrimagnetic) magnetic order is possible without further splitting of Wyckoff positions. From a symmetry perspective, the empirical prerequisites for a ferrimagnetic ground state are thus fulfilled. The resultant ground state is shown in the inset of Figure 6, with all magnetic moments aligned along the [010] direction—see Table S8. The lower temperature magnetic transition at $T_{m1}=28\pm 4$ K can likely be attributed to the flipping of the four Fe moments to be co‐aligned with the other six Fe and two Sm moments. Effectively, the upper transition corresponds to the entrance into the paramagnetic state at $T_{m2}=76\pm 4$ K—see Figure 6. Metallic character of SmFe_5_As_3_ (Figure S7e) is consistent with nonzero density of states at the Fermi level (Figure S9a), which can also be used to estimate the value of the Sommerfeld coefficient $\gamma_{DOS}=140$ mJ ${\mathrm{mol}}^{-1}$ $K^{-2}$. This is comparable to the value of $\gamma_{0}=100$ mJ ${\mathrm{mol}}^{-1}$ $K^{-2}$, extracted from a $C_{p}/T$ vs. $T^{2}$ fit at the lowest temperature (not shown)—especially if we acknowledge that the magnetic contribution is unavoidably changing the latter. The electronic band structure for the magnetic ground state of SmFe_5_As_3_, shown in Figure S9, is complex due to the high density of states at the Fermi level. The ferrimagnetic ground state breaks the twofold screw and mirror symmetries, while preserving inversion symmetry. Since the system breaks time reversal symmetry, we can expect numerous band crossings close to the Fermi level—it is important to note that both Sm 4f and Fe 3d orbitals contribute significantly at the Fermi level. Their possible topological signatures, however, would likely be obscured by the many bands crossing the Fermi level (see Figure S9b).

### 2.6 Magnetic Phase Diagram

The combination of two magnetic species in SmFe_5_As_3_ gives rise to the H−T phase diagram, summarized in Figure 6. The values of $T_{m1}$ and $T_{m2}$, extracted from magnetic susceptibility data for two different magnetic field orientations indicate that the two transitions behave differently. In particular, $T_{m1}$ is suppressed, with the exact value of the critical field being orientation‐dependent, which highlights magnetic anisotropy of SmFe_5_As_3_—see orange versus red data points. The upper transition $T_{m2}$ moves up with field. We postulate that the lower magnetic transition occurring at $T_{m1}$ is a spin reconfiguration from the ferri‐ to ferromagnetic order, as depicted in the inset of Figure 6. Of course, investigations of SmFe_5_As_3_ in higher magnetic fields are highly desirable.

## 3 Conclusion and Outlook

Among magnetostrictive materials, rare‐earth‐based systems occupy the most prominent position, with many compounds and alloys already used in everyday applications. While efficient, their scarcity and high processing costs call for a reduction in the amount of rare‐earths used, while maintaining sufficiently high efficiency. In this work, we report the discovery of a novel arsenide compound—SmFe_5_As_3_—which exhibits unusual thermal expansion behavior as a function of temperature and magnetic field. Notably, this material demonstrates giant magnetostriction, reaching values up to $2500\times 10^{-6}$, below the magnetic phase transition temperature $T_{m2}$. Our comprehensive work highlights the intricate coupling between structural, magnetic, and electronic degrees of freedom in rare‐earth‐based iron arsenides, and underscores the potential of this and related materials for uncovering new magneto‐elastic phenomena.

Single crystals of SmFe_5_As_3_ exhibit giant magnetostriction in the cryogenic temperature range, comparable to that of the commercially widespread Terfenol‐D. Furthermore, SmFe_5_As_3_ is air‐stable and relatively inexpensive compared to, for example, Tb_0.3_Dy_0.7_Fe, since the latter contains a large fraction of costly rare‐earth elements Tb (∼€800/kg) and Dy (∼€400/kg), whereas Sm (∼€15/kg), Fe, and As are significantly cheaper. In principle, the volumetric effects of SmFe_5_As_3_ could likely be further enhanced through chemical substitution or by modifying the synthesis protocol, for instance, via thin‐film growth [40, 41]. Accessing the thermo‐elastic behavior of the a‐axis of SmFe_5_As_3_ can potentially yield even more dramatic properties, paving the way for improving functionality even further.

## Conflicts of Interest

The authors declare no conflicts of interest.

## Supporting information


**Supporting File 1**: The additional references are cited in supporting information [42, 43, 44, 45, 46, 47, 48, 49, 50, 51, 52, 53, 54, 55, 56, 57, 58, 59, 60, 61, 62, 63, 64, 65, 66, 67].

**Supporting File 2**: anie71879‐sup‐0002‐SuppMat.pdf.

## Data Availability

The data that support the findings of this study are available from the corresponding author upon reasonable request.
