# Supplementary material for: Giant Magnetostriction in Ferrimagnetic SmFe5As3
Source: Angew Chem Int Ed Engl. 2026 Mar 25;65(20):e22578. doi: 10.1002/anie.202522578 (PMC13159432; doi:10.1002/anie.202522578)

# checkCIF/PLATON report

Structure factors have been supplied for datablock(s) SmFe5As3_OK_163e

THIS REPORT IS FOR GUIDANCE ONLY. IF USED AS PART OF A REVIEW PROCEDURE FOR PUBLICATION, IT SHOULD NOT REPLACE THE EXPERTISE OF AN EXPERIENCED CRYSTALLOGRAPHIC REFEREE.

No syntax errors found.    CIF dictionary    Interpreting this report

## Datablock: SmFe5As3_OK_163e

```
Bond precision:   Fe-Fe = 0.0019 A         Wavelength=0.71073

Cell:             a=7.18704(3)     b=3.85051(2)         c=9.71547(5)
                  alpha=90         beta=100.538(1)      gamma=90
Temperature:      293 K

                 Calculated                    Reported
Volume           264.329(2)                    264.329(2)
Space group      P 21/m                        P 21/m
Hall group       -P 2yb                        -P 2yb
Moiety formula   As3 Fe5 Sm                    As3 Fe5 Sm
Sum formula      As3 Fe5 Sm                    As3 Fe5 Sm
Mr               654.37                        654.36
Dx,g cm-3        8.222                         8.220
Z                2                             2
Mu (mm-1)        42.757                        42.760
F000             582.0                         582.0
F000'            585.82
h,k,lmax         11,5,14                       10,5,14
Nref             1117                          3378
Tmin,Tmax        0.288,0.343                   0.059,0.363
Tmin'            0.000

Correction method= # Reported T Limits: Tmin=0.059 Tmax=0.363
AbsCorr = MULTI-SCAN
```

```
Data completeness= 3.024          Theta(max)= 33.106

                                                   wR2(reflections)=
R(reflections)= 0.0365( 3051)                      0.1171( 3378)

S = 1.137                 Npar= 57
```

---

The following ALERTS were generated. Each ALERT has the format
**test-name_ALERT_alert-type_alert-level.**
Click on the hyperlinks for more details of the test.

---

### Alert level C

```
PLAT911_ALERT_3_C Missing FCF Refl Between Thmin & STh/L=    0.600          3 Report
                0  2  0,  -2  1  2,   1  3  5,
PLAT918_ALERT_3_C Reflection(s) with I(obs) much Smaller I(calc) .          1 Check
                0 -4  0,
```

---

### Alert level G

```
PLAT004_ALERT_5_G Polymeric Structure Found with Maximum Dimension          3 Info
PLAT012_ALERT_1_G No      _shelx_res_checksum Found in CIF ......     Please Check
PLAT142_ALERT_4_G s.u. on b - Axis Small or Missing .............    0.00002 Ang.
PLAT143_ALERT_4_G s.u. on c - Axis Small or Missing .............    0.00005 Ang.
PLAT199_ALERT_1_G Reported _cell_measurement_temperature ..... (K)        293 Check
PLAT200_ALERT_1_G Reported   _diffrn_ambient_temperature ..... (K)        293 Check
PLAT700_ALERT_1_G Superseded _atom_site_symmetry_multiplicity name    Please Check
PLAT794_ALERT_5_G Tentative Bond Valency for Sm1       (III)     .       3.00 Info
PLAT870_ALERT_4_G ALERTS Related to Twinning Effects Suppressed ..          ! Info
PLAT883_ALERT_1_G Absent Datum for _atom_sites_solution_primary ..     Please Do !
PLAT912_ALERT_4_G Missing # of FCF Reflections Above STh/L=  0.600         54 Note
PLAT913_ALERT_3_G Missing # of Very Strong Reflections in FCF ....          1 Note
                0  2  0,
PLAT931_ALERT_5_G CIFcalcFCF  Twin Law ( 1 0-1)         Est.d BASF       0.48 Check
PLAT969_ALERT_5_G The 'Henn et al.' R-Factor-gap value ..........      11.581 Note
             Predicted wR2: Based on SigI**2  1.01 or SHELX Weight 10.30
```

---

```
   0 ALERT level A = Most likely a serious problem - resolve or explain
   0 ALERT level B = A potentially serious problem, consider carefully
   2 ALERT level C = Check. Ensure it is not caused by an omission or oversight
  14 ALERT level G = General information/check it is not something unexpected

   5 ALERT type 1 CIF construction/syntax error, inconsistent or missing data
   0 ALERT type 2 Indicator that the structure model may be wrong or deficient
   3 ALERT type 3 Indicator that the structure quality may be low
   4 ALERT type 4 Improvement, methodology, query or suggestion
   4 ALERT type 5 Informative message, check
```

---

---

It is advisable to attempt to resolve as many as possible of the alerts in all categories. Often the minor alerts point to easily fixed oversights, errors and omissions in your CIF or refinement strategy, so attention to these fine details can be worthwhile. It is up to the individual to critically assess their own results and, if necessary, seek expert advice.

---

**PLATON version of 26/09/2025; check.def file version of 20/09/2025**

---

# duplicate check

**No duplication found**

---

**Datablock SmFe5As3_OK_163e** - ellipsoid plot

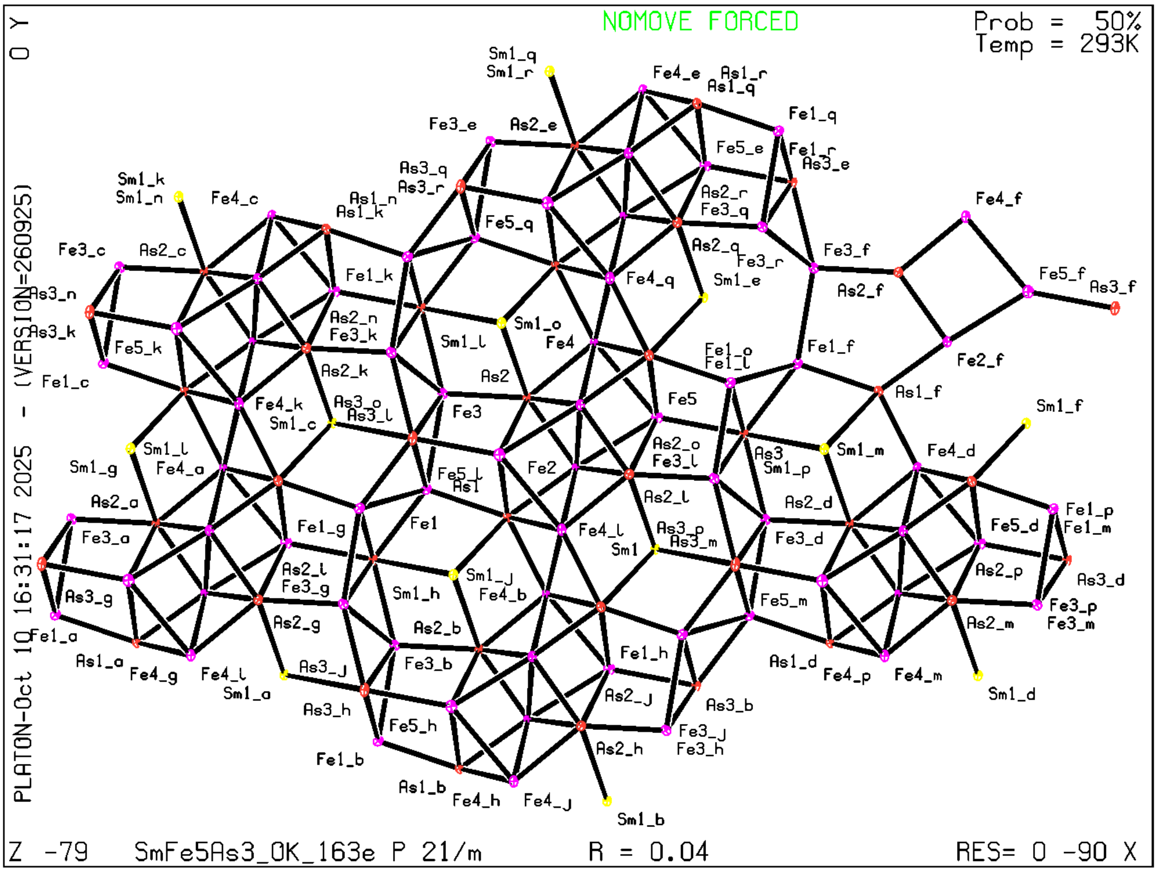

Supplement: Supplementary file 1 — Supporting File 1: The additional references are cited in supporting information [42, 43, 44, 45, 46, 47, 48, 49, 50, 51, 52, 53, 54, 55, 56, 57, 58, 59, 60, 61, 62, 63, 64, 65, 66, 67]. [file ANIE-65-e22578-s001.zip › anie71879-sup-0002-Data/yupr_2525_SmFe5As3_checkcif.pdf]
