# Supplementary material for: Giant Magnetostriction in Ferrimagnetic SmFe5As3
Source: Angew Chem Int Ed Engl. 2026 Mar 25;65(20):e22578. doi: 10.1002/anie.202522578 (PMC13159432; doi:10.1002/anie.202522578)
Supplement: Supplementary file 2 — Supporting File 2: anie71879‐sup‐0002‐SuppMat.pdf. [file ANIE-65-e22578-s002.pdf]

Supporting Information

1. Synthesis

Sample preparation was carried out in an argon-filled glovebox to prevent oxidation and moisture contamination. Single crystals of the novel ternary arsenide $SmFe_5As_3$ were synthesized using the metal-flux method. High-purity elemental samarium (sheets, ChemPur, 99.98%), iron (powder, ChemPur, 99.9%), arsenic (pieces, Alfa Aesar, 99.9999%), and bismuth (granules, ChemPur, 99.9999%) were weighed in a molar ratio of 1:5:3:20. The mixture was placed in an alumina Canfield-Svanidze crucible set,[42] which was then sealed in an evacuated quartz ampule. The ampule was heated to 1100°C and held at this temperature for 28 hours. Subsequently, the furnace was slowly cooled to 700 °C over a period of 120 hours, followed by an isothermal hold at this temperature for 120 hours to promote crystal formation. After the reaction, the bismuth flux was removed by centrifugation, yielding well-formed crystals of $SmFe_5As_3$. The crystals have a needle-like shape — see Fig. 1(b) — with the long edge corresponding to the [010] axis. These crystals have elemental Bi on the surface and in the trenches, are mechanically fragile, yet stable against air and moisture.

2. Crystal structure determination

Phase identification was performed using laboratory equipment (Huber G670 image plate Guinier camera, CuKα_1 radiation, $\lambda = 1.54056$ Å, Ge-monochromator). For the single crystal investigations, specimens obtained by Bi flux growth were used. X-ray experiments were performed using relatively thin (~20 μm) but long (~200 μm) needle-shaped crystals, which were mounted on the thin glass capillary with a sharp tip. Single crystal X-ray diffraction data were collected on a Rigaku AFC7 diffractometer equipped with a Saturn 724+ CCD detector, using MoKα radiation ($\lambda = 0.71073$ Å). Data reduction was performed by using CrysAlis^{PRO}SM software.[43] After careful analysis of the reconstructed reciprocal space (see Fig. S5(c)), it clearly indicates the building of twinned agglomerates. The orientation of the twinning domains is described by the twinning law [-0.3547 0 0.6452 0 0$\bar{1}$0 1.3548 0 0.354], which corresponds to the rotation by 180° about the [101] vector in direct space. After defining the distribution of the domains, the first twin domain was manually specified, whereas the second component was automatically generated using appropriate software. Integration was performed simultaneously for both components, and as a result, an HKLF5 file was obtained. Lattice parameters, symmetry, composition, as well as powder diffraction pattern pointed that the investigated phase is isostructural with UFe_5As_3.[18] The atomic coordinates of the latter were used as starting values for crystal structure refinement. Refinement with anisotropic displacement parameters for all atomic positions resulted in residuals $R_1 = 0.0365$ and $wR_2 = 0.1171$ and twin component ratio 0.563(2) : 0.437. For the final runs (WinGX suite of programs[44]) lattice parameters obtained from the high-resolution powder diffraction data (see Fig.S1) were used (309 reflections, 2θ range = $3.24 - 25.14°$, $\lambda = 0.35431$ Å). The individual peak positions were extracted by profile fitting and corrected by LaB_6 as an internal standard (WinCSD software package[45]).

3. Temperature-dependent X-ray diffraction

To investigate the correlation between structure evaluation and physical properties, a series of high-resolution X-ray powder measurements was performed (synchrotron source, ID22 at the ESRF, Grenoble, $\lambda = 0.35431$ Å).[46] For this purpose, selected single crystals of $SmFe_5As_3$ were ground into fine powder and loaded into 0.3 mm diameter silica-glass capillaries. Data collection was performed by variable temperature programs (from room temperature to 100 K with a step of 50 K, from 100 K to 40 K with a step of 5 K, and from 40 K to 10 K with a step of 3 K) – see Fig. 2. To detect possible hysteresis, measurements were performed by cooling and heating. By the conventional setup of the beamline, no phase structural transitions were detected despite the effects observed below 60 K during measurement of physical properties. Only small bumps in the pattern recorded at 10 K indicated such structural changes (Fig. S2). The absence of a clear signal indicates non-compliance of the real temperature with the set value due to the sample heating by the intense synchrotron beam. To reduce this effect, the attenuator was used. This allowed us to clearly indicate structural changes at the lowest temperature (see Fig. S2). Full profile refinement was performed for all collected patterns by using the Jana2006 crystallographic computing system.[47] The refined parameters included the overall scale factor, unit cell dimensions, atomic positions, anisotropic displacement parameters, and background, were modeled using a Chebyshev polynomial. Goodness-of-fit indicators (e.g., R_{wp}, R_p, χ^2) were monitored to ensure reliability and stability of the refinements.

Lattice parameters obtained from XRD measurements were used to reconstruct the temperature-dependent unit-cell volume $V(T)$ (see Fig. 2(b) and (c)). The $V(T)$ was modeled using a second-order Grüneisen approximation for the zero-pressure equation of state:[24–29]

$$V(T) = V_0 + \frac{V_0\, U(T)}{Q - b\, U(T)}. \quad (1)$$

The lattice internal energy $U(T)$ was evaluated within the Debye model,

$$U(T) = 9Nk_BT\left(\frac{T}{\theta_D}\right)^3 \int_0^{\theta_D/T} \frac{x^3}{e^x - 1}\, dx, \quad (2)$$

where k_B is Boltzmann's constant, N is the number of atoms per unit cell, and θ_D is the Debye temperature. The result is shown in Fig. S3. The resultant parameters are $V_0 = 261.53$ Å^3, $Q = 7.0 \times 10^{-18}$ J, $b = 2.9$ and $\theta_D = 311$ K. These values provide a quantitative description of the observed volume evolution with temperature within the quasi-harmonic framework. Below 80 K volume shrinks and at the lowest temperature, the difference between the evaluated volume and the experimental one $\omega = \Delta V/V = 0.12\%$.

4. Scanning transmission electron microscopy

Cross-sectional STEM specimens were prepared by the standard focused ion beam (FIB) lift-out procedure on a Thermo Fisher Scientific Helios G5 FIB. STEM images were collected

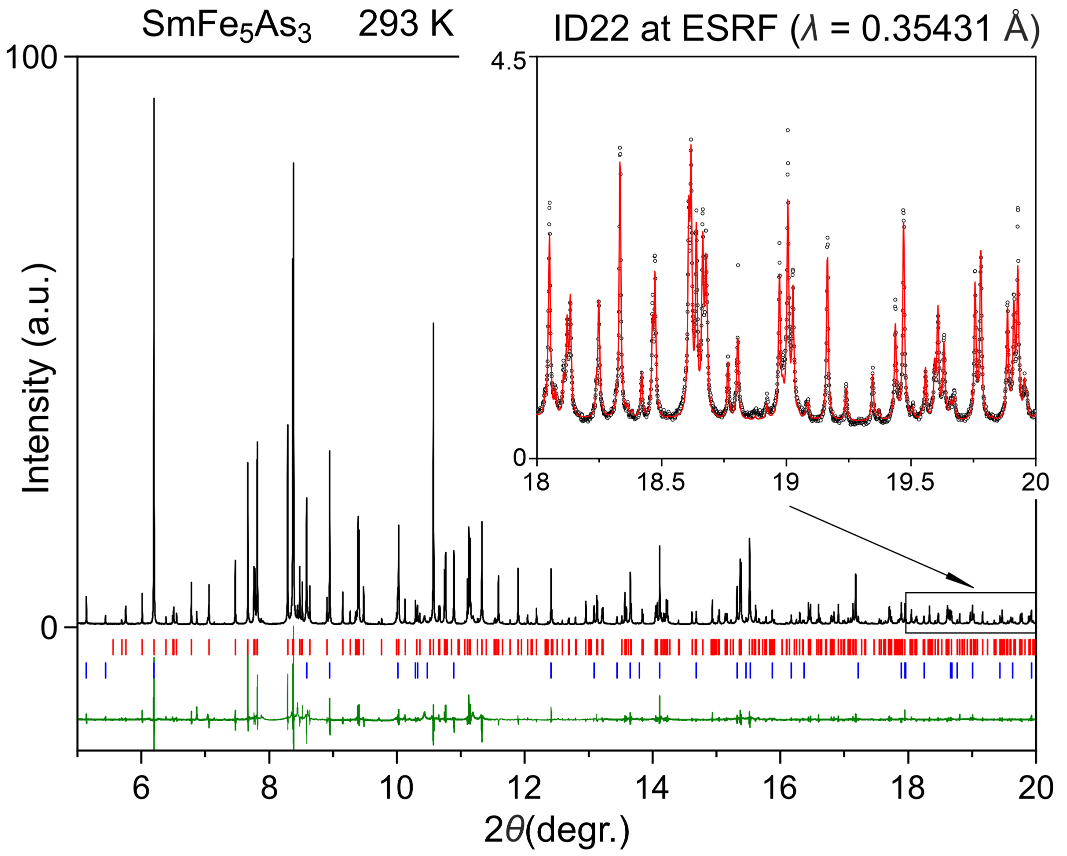

Figure S1. Powder X-ray diffraction pattern of $SmFe_5As_3$ measured with synchrotron radiation at room temperature: black solid line – experimental pattern; red ticks – peak positions of $SmFe_5As_3$; blue ticks – peak positions of Bi used as flux for crystal growth; green line – the difference between measured and calculated intensities. The inset shows a selected region to demonstrate an excellent agreement between experimental (black circles) and calculated intensities (red solid line) for weak reflections at higher $1/d$. The small number (top left of inset) gives the relative intensity compared to that of the strongest reflection.

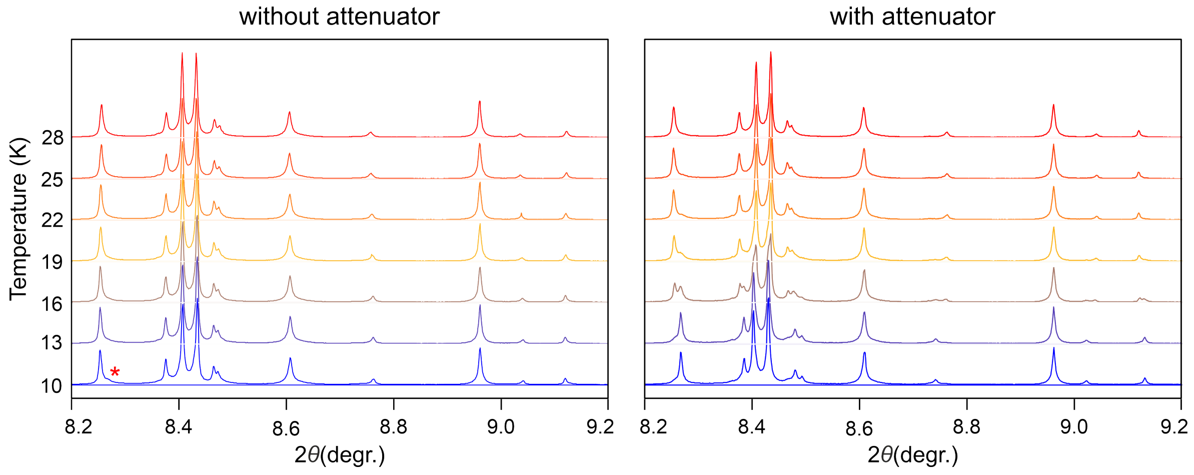

Figure S2. Selected regions of the diffraction patterns from synchrotron powder X-ray diffraction, obtained from powdered single crystals of $SmFe_5As_3$ without (left panel) and with (right panel) attenuator, recorded in the range 10–28 K. There are virtually no signs of a new modification, with the exception of the bump marked with an asterisk in the 10 K pattern (left). Clear evidence of a new modification at 10 and 13 K, as well as the presence of two phases at 16 K, can be seen on the right part of the figure.

Table S1. Crystallographic data of $SmFe_5As_3$.[a]

Composition	$SmFe_5As_3$
Structure type	UCr_5P_3
Space group	$P2_1/m$
Z	2
Pearson symbol	$mP18$
Lattice parameters [b]	
a, Å	7.18704(3)
b, Å	3.85051(2)
c, Å	9.71547(5)
β, Å	100.538(1)
V, Å^3	264.329 (2)
Calc. density, g cm^{-3}	8.222
Radiation	MoKα, $\lambda = 0.71073$ Å
2θ range, °	4.2 – 66.2
Range in h, k, l	$-10 \leq h \leq 10$
	$-5 \leq k \leq 5$
	$-14 \leq l \leq 14$
Absorption coeff., mm^{-1}	42.76
Absorption correction	multi-scan
T(max)/T(min)	6.14
$N(hkl)$ measured [c]	3378
$N(hkl)$ observed	3051
Observation criteria	$F(hkl) \geq 4\sigma\ [F(hkl)]$
Twin component ratio	0.563(2) : 0.437
Refined parameters	57
R_1; wR_2	0.0365; 0.1171
Residual peaks, e Å^{-3}	2.40 / –3.11

[a]The cif file has been deposited at the Cambridge Crystallographic Data Center (CSD number 2494555) and contains the supplementary crystallographic data for this paper. These data can be obtained free of charge via website, by email, or by contacting the Cambridge Crystallographic Data Center, 12 Union Road, Cambridge CB2 1EZ, UK.
[b]Lattice parameters obtained from synchrotron powder diffraction data (309 reflections, $2\theta = 3.24 - 25.14°$, $\lambda = 0.35431$ Å, LaB_6 as an internal standard).
[c]The data set was not averaged due to the twinning.

Table S2. Atomic coordinates and equivalent displacement parameters (in Å^2) of $SmFe_5As_3$.

Atom	Wyckoff site	x/a	y/b	z/c	U_{eq}
Sm1	2e	0.19302(8)	¼	0.78383(6)	0.0063(2)
Fe1	2e	0.0835(3)	¼	0.09133(19)	0.0091(3)
Fe2	2e	0.3817(2)	¼	0.51140(17)	0.0060(3)
Fe3	2e	0.4614(3)	¼	0.08508(19)	0.0093(4)
Fe4	2e	0.8703(2)	¼	0.49607(18)	0.0068(4)
Fe5	2e	0.6831(3)	¼	0.7172(2)	0.0096(4)
As1	2e	0.09912(18)	¼	0.34074(13)	0.0062(3)
As2	2e	0.56774(17)	¼	0.33237(13)	0.0061(3)
As3	2e	0.74764(18)	¼	0.97666(13)	0.0083(3)

Table S3. Interatomic distances in $SmFe_5As_3$.

Atoms		δ, Å	Atoms		δ, Å	Atoms		δ, Å
Sm1–	2As2	2.9407(9)	Fe3–	1As2	2.384(2)	As2–	1Fe2	2.380(2)
	2As1	2.941(1)		2As3	2.448(1)		1Fe3	2.384(2)
	2As3	2.990(1)		1As3	2.477(2)		2Fe2	2.437(1)
	2Fe1	3.164(1)		2Fe3	3.661(2)		1Fe4	2.448(2)
	1Fe2	3.187(2)		1Fe1	2.727(3)		2Fe5	2.621(2)
	1Fe3	3.197(2)		2Fe5	3.034(2)		2Sm1	2.9407(9)
	2Fe3	3.216(2)		1Sm1	3.197(2)	As3–	2Fe1	2.431(1)
	1Fe1	3.226(2)		2Sm1	3.216(2)		2Fe3	2.448(1)
	1Fe4	3.290(2)	Fe4–	1As1	2.427(2)		1Fe1	2.467(2)
	2Fe4	3.295(1)		1As2	2.448(2)		1Fe3	2.477(2)
Fe1–	1As1	2.405(2)		2As1	2.478(1)		1Fe5	2.479(2)
	2As3	2.431(2)		2Fe2	2.635(2)		2Sm1	2.990(1)
	1As3	2.467(2)		2Fe4	2.671(2)			
	1Fe3	2.727(3)		1Fe5	2.735(2)			
	2Fe1	2.742(3)		1Sm1	3.290(2)			
	2Fe5	2.973(2)		2Sm1	3.295(1)			
	2Sm1	3.164(1)	Fe5–	1As3	2.477(2)			
	1Sm1	3.226(2)		2As1	2.607(2)			
Fe2–	1As1	2.375(2)		2As2	2.621(2)			
	1As2	2.380(2)		1Fe2	2.666(3)			
	2As2	2.437(1)		1Fe4	2.735(2)			
	2Fe2	2.605(2)		2Fe2	2.911(2)			
	2Fe4	2.635(2)		2Fe1	2.973(2)			
	1Fe5	2.666(3)		2Fe3	3.034(2)			
	2Fe5	2.911(2)	As1–	1Fe2	2.375(2)			
	1Sm1	3.187(2)		1Fe1	2.405(2)			
				1Fe4	2.427(2)			
				2Fe4	2.478(1)			
				2Fe5	2.607(2)			
				2Sm1	2.941(1)			

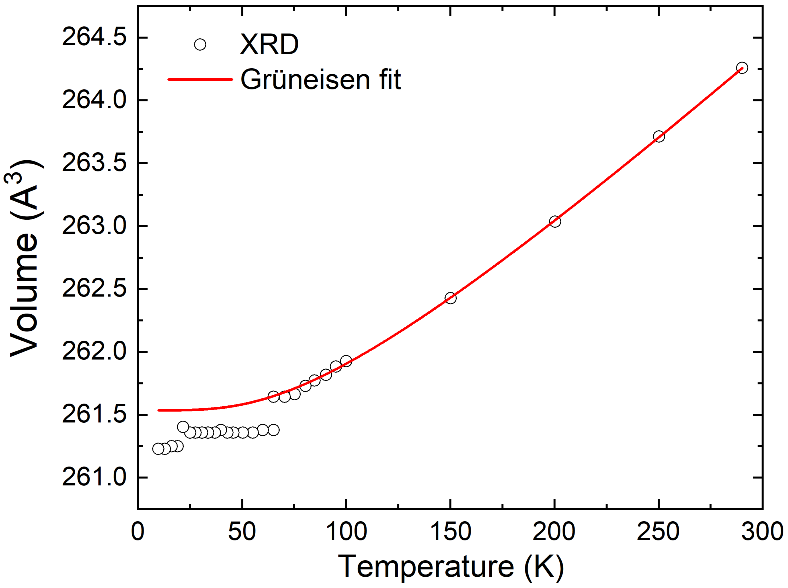

Figure S3. Temperature dependence of the unit cell volume $V(T)$ of $SmFe_5As_3$ together with the Grüneisen fitting performed above $T = 80$ K.

in high-angle annular dark-field (HAADF) configuration on a double aberration-corrected JEOL ARM 300F operating at 300 kV with a probe convergence angle of 30 mrad. Elemental mapping by electron energy loss spectroscopy (EELS) was performed on a Thermo Fisher Scientific Spectra 300 X-FEG operating at 300 kV with a 21 mrad probe convergence angle. EELS data was collected on a Thermo Fisher Scientific Iliad Ultra STEM system with a Zebra EELS detector, using a single energy shift and a dispersion of 0.42 eV/channel. Spectrum images in Fig. S4 were acquired using a probe current of 200 pA with a 1 ms per pixel dwell time, binned by 2 in both spatial dimensions post-acquisition to enhance the signal-to-noise ratio. In addition to elemental mapping, EELS also records the spectral fine structure suitable for chemical or valence analysis of the constituent elements: Fig. S6 shows the integrated EEL spectra across a typical core-loss range of ~650-1500 eV with magnified views of the Fe-$L_{2,3}$, Sm-$M_{4,5}$, and As-$L_{2,3}$ edges. The integrated spectrometer and post-specimen optics of the Iliad STEM system also enables collection at much higher energy losses,[48] extending even to the As-K edge with an 11.867 keV onset, here collected using a 200 pA probe with total acquisition time of ~250 seconds.

5. Analysis of magnetic properties

Magnetic properties were examined using a Quantum Design (QD) Magnetic Property Measurement System in the temperature range of 1.8–300 K and under various applied magnetic fields. Anisotropic measurements of $SmFe_5As_3$ single crystals are summarized in Fig. S7. Given crystal morphology, measurements have been carried out with the field perpendicular and parallel to the long edge of the needles, i.e. *b*-axis. AC electrical resistivity measurements were performed on a QD Physical Property Measurement System, using a standard four-probe technique at temperatures between $T = 2$ and 300 K. Due to the fragility of the $SmFe_5As_3$ crystals, coupled with the Bi inclusion (see Fig. 1(b)), a micro-scale device was prepared out of a single crystal using plasma focused-ion-beam (FIB).[49] The details of the procedure of micro device preparation is described elsewhere.[49–51] AC electrical resistivity measurements were performed on a QD PPMS, using a standard four-probe technique at temperatures between $T = 0.4$ and 300 K in various magnetic fields. A current pulse of 0.01 mA with frequency 93 Hz for 1 s was applied along the *b*-axis. The specific heat data were collected on a QD PPMS from 0.4 K to 10 K and under various applied magnetic fields. The temperature dependence of the specific heat $C_P(T)$ of $SmFe_5As_3$ (Fig. 3(c)) is characteristic of intermetallic systems, with a dominant phononic background corresponding to a Debye temperature on the order of room temperature. Pronounced λ-shaped features indicate magnetic phase transitions, consistent with anomalies observed in other physical properties.

6. X-ray absorption spectroscopy and Kerr microscopy

X-ray absorption near edge spectroscopy (XANES) data at the Sm L_3 edge were acquired at the ID12 beamline (ESRF – The European Synchrotron, Grenoble, France). A polycrystalline sample, consisting of an assembly of small randomly oriented needles, was glued on an aluminum holder. The data collected at room temperature were recorded using total fluorescence yield detection mode and were corrected for re-absorption effect. The size of the incident X-ray beam was tuned to probe the largest area of the sample. For the sake of comparison, the spectrum was normalized using standard procedures.

Magnetic domains on the surface of single crystalline clusters of $SmFe_5As_3$ were investigated at $T = 50$ K using longitudinal Kerr microscopy under in-plane magnetic field application.[52] The observed domains – see the inset of Fig. 3(c) – exhibit a stripe morphology, indicating the presence of a well-defined easy axis oriented within, or slightly tilted from, the sample plane in these grains.[53] This observation suggests that $SmFe_5As_3$ possesses uniaxial magnetic anisotropy, with the easy axis oriented orthogonal to the [010] (*b*-axis) crystallographic direction.

By tracing the gray level at the surface of the $SmFe_5As_3$ sample, the transition from the para- to ferromagnetic state can be followed upon cooling. As seen in Fig. S8, the onset of the transition occurs already at slightly higher temperatures than T_{m2}, derived from bulk magnetization measurements. This behavior is typical for such transitions, which often nucleate at the surface – this might also be influenced by surface damage introduced during polishing.

7. Thermal expansion

Thermal expansion coefficients were determined from the temperature dependence of the lattice parameters. The main axes of the thermal expansion coefficient tensors above and below the phase transition were obtained using the method delineated in the reference.[54] The unit strain tensor is based on the relative changes per degree Kelvin. Due to the monoclinic symmetry, the main axes of the thermal expansion tensor in the *ac*-plane are not aligned with the *a*- and *c*-axes. Above the phase transition, the diagonalized unit strain tensor has ϵ_{22} aligned along the *b*-axis, ϵ_{11} at 136.8° from the *a*-axis, and ϵ_{33} at 46.8° from the *a*-axis. Note that the unit strain values given are $\epsilon_{ii} \times 10^6\,\mathrm{K}^{-1}$.

$$\begin{pmatrix} 17.5 & 0 & 0 \\ 0 & 16.5 & 0 \\ 0 & 0 & 13.5 \end{pmatrix}$$

Below the phase transition the unit strain is obtained between the temperatures of 30 K and 90 K. The tensor component ϵ_{11} is aligned 2.6° from the *a*-axis, ϵ_{22} is along the *b*-axis, and ϵ_{33} is 92.6° from the *a*-axis.

$$\begin{pmatrix} 203.8 & 0 & 0 \\ 0 & -82.1 & 0 \\ 0 & 0 & -99.3 \end{pmatrix}$$

The unit strain tensor is reoriented below the phase transition, and the unit strain values are significantly larger than above the phase transition. The large contraction upon cooling that is observed along the *a*-axis has a commensurate expansion perpendicular to it.

8. Capacitance dilatometry

The temperature and magnetic field dependence of the linear thermal expansion, $\Delta L/L_0$, were investigated using a Quantum Design PPMS DynaCool platform equipped with a stress-free capacitance dilatometer from Kuechler Innovative

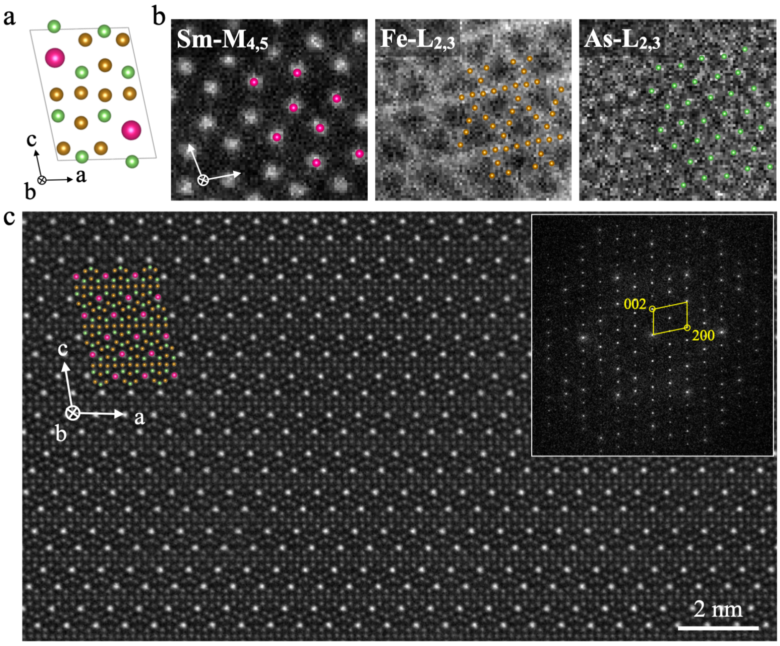

Figure S4. (a) Crystal model of $SmFe_5As_3$ viewed along the [010] projection and (b) atomic-resolution STEM-EELS elemental maps of Sm (magenta), Fe (orange), and As (green) along the same projection with overlaid models of each sublattice. (c) Atomic-resolution HAADF-STEM image along the same crystalline projection, with inset Hann-windowed fast Fourier transform (FFT).

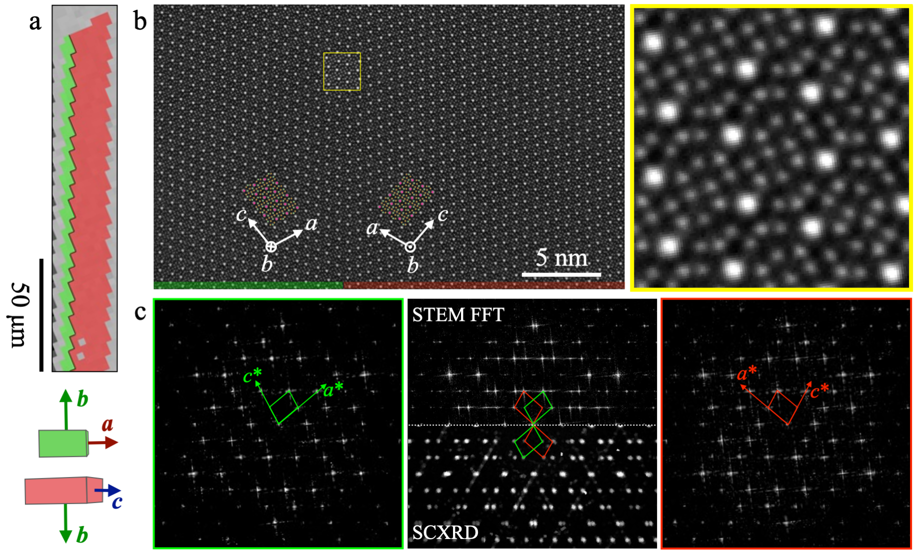

Figure S5. (a) EBSD of $SmFe_5As_3$ single crystals (left) reveals local variation in crystal a and c axis orientations, while the b-axis is always aligned along the long edge of the crystals as picture by the colored models corresponding to different regions. (b) A similar twin boundary is also observed by HAADF-STEM imaging (middle), revealing an abrupt transition (yellow line) between the two local orientations marked by overlaid atomic models. The inset (magnified at right) is a local average of cropped regions at the interface, showing the detailed atomic structure at the boundary. (c) Fast Fourier transforms (FFTs) from regions on either side of the boundary marked by green and red further confirm the two orientations; an FFT spanning both sides of the boundary reproduces the bulk single crystal X-ray diffraction (SCXRD) pattern (middle).

Table S4. Symmetric strain tensor obtained from unit cell parameters at $T = 90$ K and $T = 295$ K (strain values $\times 10^4$), b-axis along z.

$$\begin{pmatrix} 30.56 & 3.95 & 0 \\ 3.95 & 33.04 & 0 \\ 0 & 0 & 33.87 \end{pmatrix}$$

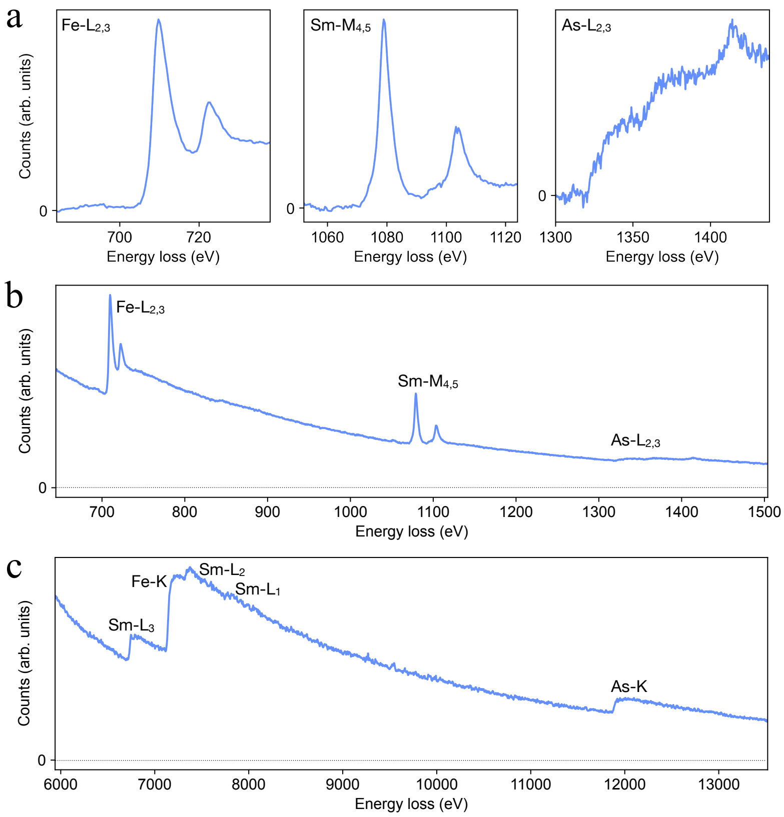

Figure S6. Electron energy loss spectra from $SmFe_5As_3$, including (a) background-subtracted core-loss edges used to produce the elemental maps in Fig. S4 and (b) the corresponding raw integrated spectrum. (c) The bottom panel shows high energy loss edges Sm-L_3 (6716 eV), Fe-K (7112 eV), Sm-L_2 and Sm-L_1 (7312 and 7737 eV, respectively), and As-K (11867 eV).

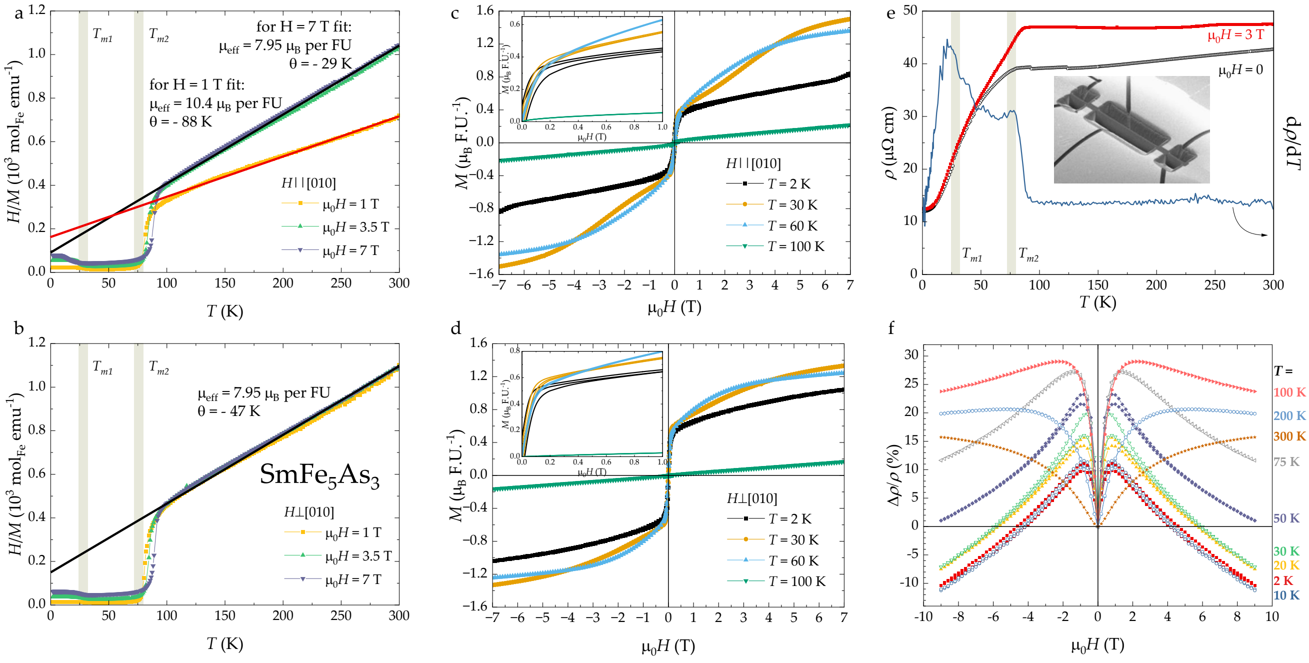

Figure S7. Magnetic properties of $SmFe_5As_3$: (a) and (b) For both directions of the applied magnetic field, strong anomalies accompany entrance into magnetic state at $T_{m2} = 76 \pm 4$ K, with a less pronounced feature around the lower magnetic transition $T_{m1} = 28 \pm 4$ K. The effective magnetic moments, extracted from the Curie-Weiss fit above $T = 150$ K amount to 7.95 – 10.4 μ_B per FU. All of the Weiss temperatures are indicating antiferromagnetic coupling in $SmFe_5As_3$, with value between –29 K and –88 K. (c) and (d): Field-dependent isotherms are consistent with the assigned magnetic configurations, additionally showing some metamagnetic transitions at higher fields. (e) Temperature-dependent resistivity of $SmFe_5As_3$ in $H = 0$ (black) and $H = 3$ T (red), measured on a micro-scale device (inset). Right scale: derivative of resistivity (blue) with respect to temperature shows features close to T_{m1} and T_{m2}. (f) Field-dependent resistivity, taken at various temperatures on the micro-scale device, shown in the inset of panel (e).

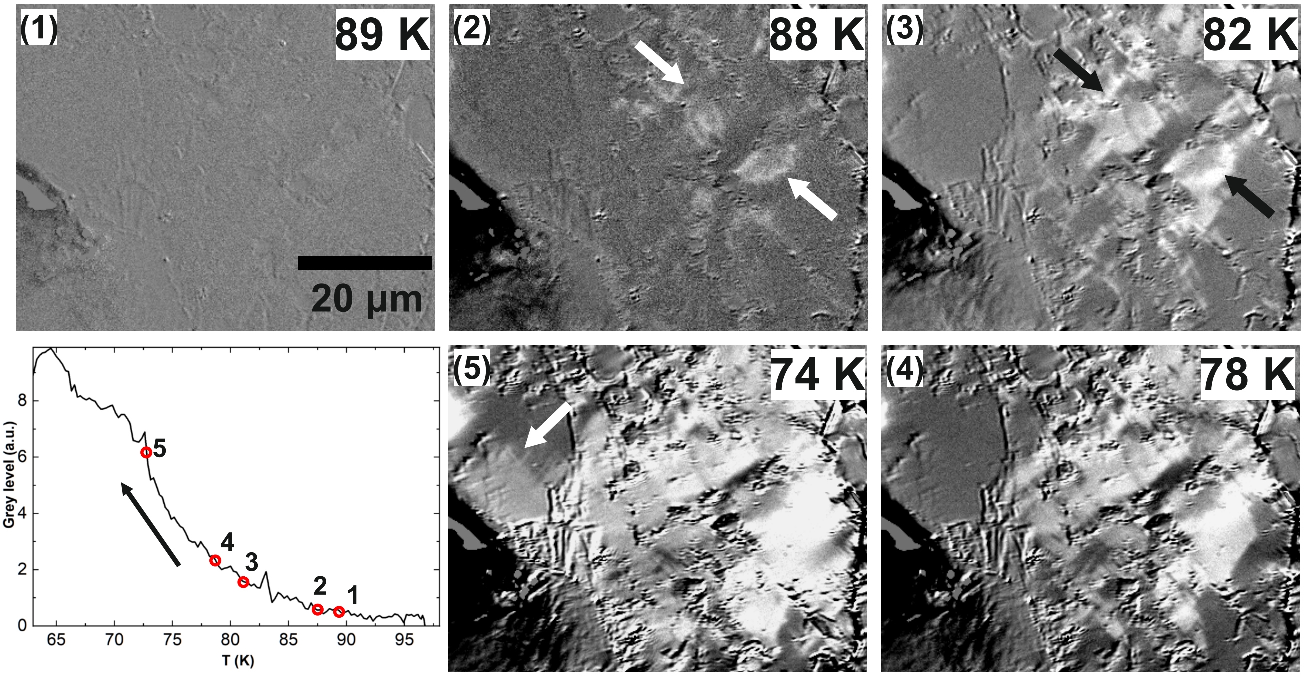

Figure S8. The transition of the $SmFe_5As_3$ sample from para- to ferromagnetic state upon reaching T_{m2} during cooling, observed at the sample surface. The appearance of bright areas indicates the regions, which become ferromagnetic.

Table S5. Diagonalized unit strain tensor above the 65 K phase transition $\epsilon_{ij} \times 10^6$ [K^{-1}].

$$\begin{pmatrix} 17.5 & 0 & 0 \\ 0 & 16.5 & 0 \\ 0 & 0 & 13.5 \end{pmatrix}$$

Table S6. Symmetric strain tensor obtained from unit cell parameters at 30 and 90 K (strain values $\times 10^4$), b-axis along z.

$$\begin{pmatrix} 119.2 & -23.6 & 0 \\ -23.6 & -56.5 & 0 \\ 0 & 0 & -49.3 \end{pmatrix}$$

Table S7. Diagonalized unit strain tensor below the 20 K phase transition $\epsilon_{ij} \times 10^6$ [K^{-1}].

$$\begin{pmatrix} 203.8 & 0 & 0 \\ 0 & -82.1 & 0 \\ 0 & 0 & -99.3 \end{pmatrix}$$

Measurement Technology. This dilatometer offers an absolute resolution of $\Delta L = 0.01$ Å and a high data point density, enabling detailed insight into structural changes associated with various phase transitions. A comprehensive description of the capacitance dilatometry technique and data processing procedures is available in Ref.[55]. Measurements were performed along the direction parallel to [010], down to 2 K and in magnetic fields up to 14 T, applied along the same axis. Despite multiple attempts, reliable thermal expansion data along the [010] direction could not be obtained due to the fragility of the single crystals.

9. Ab initio calculations

Ab initio calculations were conducted using density functional theory (DFT) and the VASP code[56–59] based on the projector augmented wave (PAW) method.[60,61] Perdew-Burke-Ernzerhof (PBE) functional[62,63] and the generalized gradient approximation were used to model exchange-correlation effects. Standard VASP pseudopotentials were used (Sm, Fe, and As) for treatment of core electrons. On top of that, 200 bands were simulated.

Strongly-correlated samarium f-electrons were treated via adding a Hubbard U term (DFT+U) using Dudarev's effective $U - J$ approach[64] with a value of $U - J = 6$ eV consistent with previous studies of Sm^{3+} ion.[65] Constrained local moments approach available in VASP[66,67] was later used to facilitate convergence of the ground state, although it was not used in the initial magnetic configuration space exploration. Both direction and sign of the moments were constrained (flag $I\_CONSTRAINED\_M = 4$). The penalty energy was set to $E_p = 1$ eV. The plane-wave cutoff energy was set to 400 eV and the convergence criterion was set to $1 \cdot 10^{-7}$ eV. K-point mesh optimization based on symmetry was disabled.

Table S8. Magnetic moments (spin, orbital and total) of the DFT-converged ground state for $SmFe_5As_3$. All moments are aligned along the b-axis.

Atom	m_s [μ_B]	m_{orb} [μ_B]	m_{tot} [μ_B]
Sm1	+5.15	−2.96	+2.19
Fe1	−1.62	−0.06	−1.68
Fe2	+1.85	−0.06	+1.91
Fe3	−1.85	−0.07	−1.92
Fe4	+2.07	−0.07	+2.14
Fe5	+2.31	−0.09	+2.40
total	+7.91	−2.87	+5.04

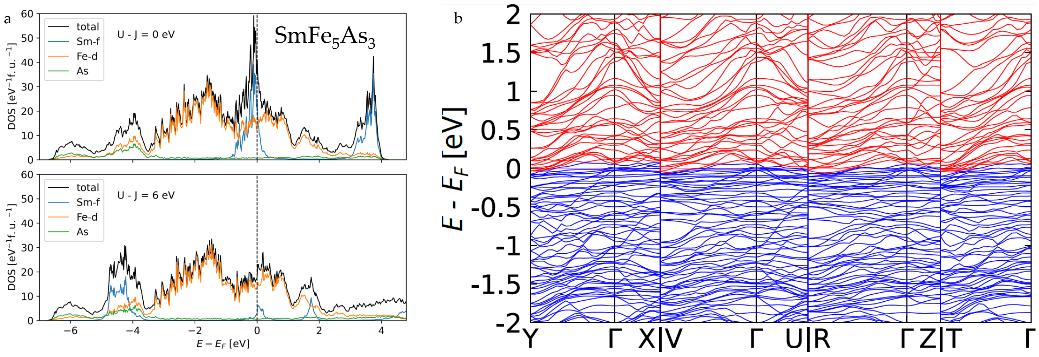

Figure S9. (a) The orbital-resolved density of states for Hubbard $U - J = 0$ eV and $U - J = 6$ eV. Both Sm f- and Fe d−states have a significant contribution at the Fermi level, yielding $\gamma_{DOS} = 52$ mJ mol^{-1} K^{-2}. (b) Band structure of $SmFe_5As_3$ for the magnetic ground state.
